# Integrated opisthorchiasis control through the EcoHealth/one health approach: 15 years of success and experiences with the Lawa model

**DOI:** 10.1016/j.onehlt.2025.101006

**Published:** 2025-02-28

**Authors:** Banchob Sripa, Sirikachorn Tangkawattana, Mingkwan Sangnikul

**Affiliations:** a World Health Organization Collaborating Centre for Opisthorchiasis Research and Control, Khon Kaen University, Khon Kaen, Thailand; b Department of Tropical Medicine, Faculty of Medicine, Khon Kaen University, Khon Kaen, Thailand; c Faculty of Veterinary Medicine, Khon Kaen University, Khon Kaen, Thailand; d Ban Lawa Health Promoting Hospital, Mueang Phia Subdistrict, Ban Phai District, Khon Kaen Province, Thailand

**Keywords:** Liver fluke, *Opisthorchis viverrini*, Control, One health, Lawa model, Thailand

## Abstract

*Opisthorchis viverrini* infection remains a major health problem in Northeast Thailand and the Mekong region impacting over 12 million and causing bile duct cancer. Using an EcoHealth/One Health approach at Lawa Lake in Thailand, our integrated control program achieved a substantial reduction in liver fluke prevalence from 60 % to <5 % over 15 years. Key interventions included chemotherapy, collaboratively designed health education, ecosystem modification, and community participation. Infections in intermediate hosts, *Bithynia* snails and Cyprinoid fish, are now undetectable. Improved community knowledge resulted in healthier practices. The “Lawa Model”, a recognized model for liver fluke control, is now a training hub being scaled up in Thailand and the Mekong region. This program demonstrates how One Health strategies can address complex health and ecological challenges and aligns with WHO recommendations. The success of the Lawa Model demonstrates the efficacy of integrated One Health interventions against endemic parasitic diseases.

## 1 Introduction

Liver fluke infection caused by *Opisthorchis viverrini* is a major public health concern in Southeast Asia, affecting over 10 million people in Thailand, Lao PDR, Cambodia, Vietnam, and Myanmar [1,2]. The disease is linked to consuming raw or undercooked freshwater fish harboring parasite larvae, leading to chronic hepatobiliary diseases, including cholangiocarcinoma (CCA), a deadly bile duct cancer [3]. Despite clear evidence of this link, control efforts in endemic rural areas remain fragmented and largely ineffective.

Conventional programs focused on drug treatments and education have struggled to reduce infection rates or curb CCA. Challenges include inadequate health education, entrenched cultural practices, and limited interdisciplinary collaboration [[4], [5], [6], [7], [8]]. This short report highlights the success of the Lawa Model, an integrated liver fluke control program using EcoHealth/One Health approach in Northeast Thailand over the past 15 years. Combining chemotherapy, innovative health education, environmental management, and community engagement, the model provides valuable lessons for scaling up liver fluke control regionally and globally.

## 2 Life cycle of *Opisthorchis viverrini*

**Fig. 1:**
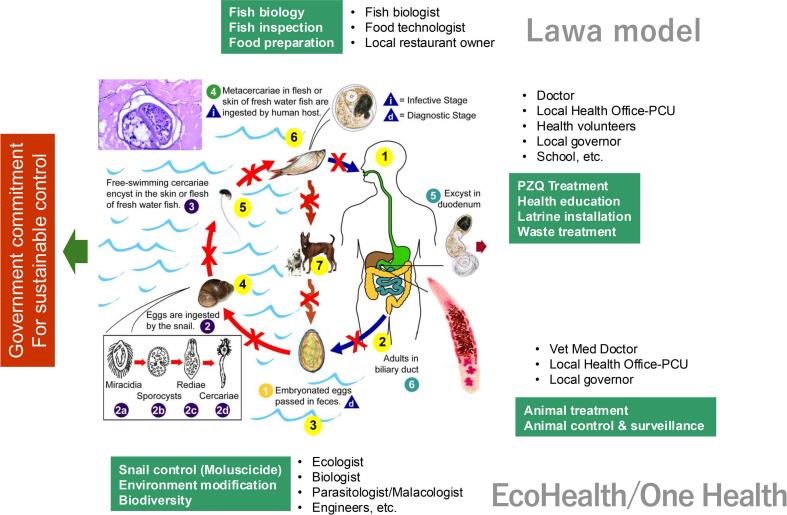
Life cycle of *Opisthorchis viverrini* and the integrated systems thinking framework of the Lawa Model (modified from Sripa et al., 2017 and https://www.cdc.gov/dpdx/opisthorchiasis/index.html).

**Fig. 2:**
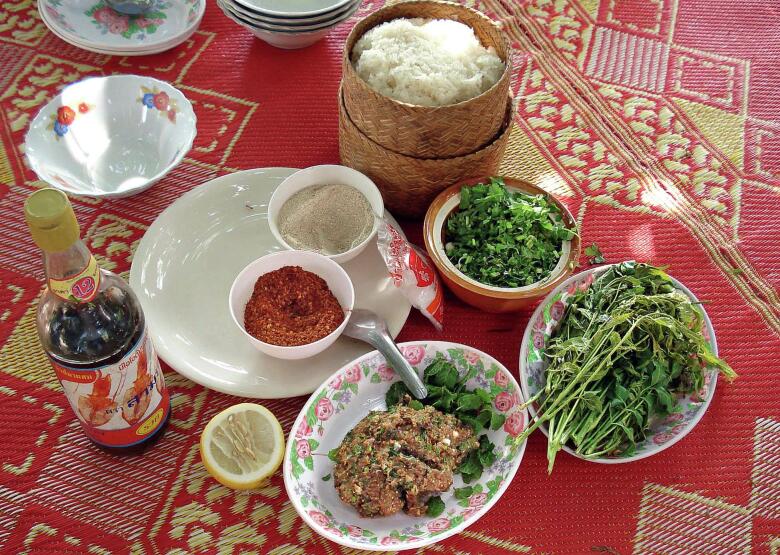
Traditional raw fish spicy salad (*koi pla*) and its typical ingredients, widely consumed by local residents.

The liver fluke *O. viverrini* has a complex life cycle (Fig. 1) [1]. Humans become infected by consuming raw or undercooked freshwater fish containing infective metacercariae, commonly through traditional dishes like *koi pla* (raw fish spicy salad), *pla som* (short-fermented fish), or improperly fermented fish (*pla ra*) (Fig. 2). After ingestion, the larvae migrate to the liver via the bile ducts, where they mature, mate, and begin producing eggs [9]. Eggs excreted in human feces reach water sources, where they are ingested by *Bithynia* snails. Inside the snails, the parasite progresses through several asexual stages-miracidium, sporocyst, redia, and cercaria-over approximately one month. The cercariae then infect fish, primarily of the Cyprinidae family, where they encyst as metacercariae within the fish tissue. Humans or animals that consume these infected fish become hosts to the mature parasites, which settle in their bile ducts and complete the life cycle. Cats and dogs serve as important reservoirs for *O. viverrini*, with infection rates in cats reaching up to 80 % in some endemic areas [10]. These animals contribute to the transmission cycle by shedding parasite eggs soil-ground and eventually into water sources through their feces. Once inside a human host, liver flukes can survive up to 26 years [11], perpetuating the cycle and increasing the risk of severe hepatobiliary diseases.

## 3 The Lawa Lake and liver fluke problems

Lawa Lake, designated as Thailand's 15th nationally important wetland, spans over 5000 acres with a maximum length of 10 km, extending from deeper waters in the north to shallower waters in the south, within the Chi River Basin in Khon Kaen Province. As a typical wetland ecosystem, the lake is home to thousands of cyprinoid fish, snails, and other diverse fauna and flora species. This biodiverse aquatic ecosystem not only supports the livelihoods of over 20 nearby villages, with a combined population exceeding 20,000 but it also facilitates the transmission of the liver fluke in this area. The high abundance of cyprinoid fish and *Bithynia* snails, which serve as intermediate hosts, sustains this transmission cycle. In addition, the people in Northeast Thailand (Isaan) are predominantly of Tai-Lao descent and maintain traditional dietary practices, including a preference for raw or undercooked meat and fish, such as *koi pla*(raw fish salad) and raw pork dishes. These cultural practices, combined with a conducive transmission environment, have significantly contributed to high liver fluke infection rates in the region. Surveys conducted in 2007–2008 found infection rates surpassing 74 % in communities around Lawa Lake, prompting the development of collaborative, community-led interventions to address this pressing public health issue [12].

## 4 The Lawa project

The Lawa Project is an integrated liver fluke research and control initiative. Initially focused on diagnosing and treating infections, the project encountered annual reinfection rates exceeding 20–30 %, prompting a pivot to an EcoHealth/One Health approach [13]. This multidisciplinary framework addresses the complex dynamics of liver fluke transmission through: 1) *Transdisciplinary integration*: harnessing expertise across diverse academic and professional domains, 2) *Stakeholder participation*: engaging local communities and government agencies in decision-making and implementation, and *Equity*: Balancing human, animal, and environmental health priorities. By targeting the interconnected life cycle of the liver fluke, the Lawa Project aims to disrupt transmission and establish sustainable, community-driven control measures grounded in One Health principles including human, animal, and environmental health. Fig. 1 highlights this integrated One Health approach.

### 4.1 Lawa model history

Over two decades, the Lawa Project addressed high liver fluke infection rates around Lawa Lake, Khon Kaen Province. Baseline studies assessed infections in humans, intermediate hosts (snails and fish), and reservoir hosts (cats and dogs), alongside ecological factors and risky behaviors like eating raw fish [12]. Collaborative efforts involved academics from Khon Kaen University's Faculties of Medicine, Veterinary Medicine, Agriculture, and Public Health, alongside community leaders, schools, health centers, regional education offices, village health volunteers, and Subdistrict Administrative Organizations. These stakeholders co-developed the “Lawa Model” through forums and focus groups, establishing a sustainable and integrated liver fluke control strategy [14]. The Lawa Model initially had no dedicated grant support, relying solely on a seeding grant from Khon Kaen University for multidisciplinary liver fluke research. As the control model took shape, we secured funding from the Thailand Research University Network and the National Research Council of Thailand. Through international collaborations, we obtained sub-contracted grants from Grand Challenges Canada and the International Development Research Centre (IDRC, Canada). Additionally, community outreach efforts were significantly facilitated by piggybacking on National Institutes of Health (NIH) grants (ICIDR and TMRC) focused on the pathogenesis of liver fluke-induced cancer in Thailand.

### 4.2 Implementation framework

The Lawa Project fostered community empowerment and collaboration with schools to ensure local ownership of liver fluke control efforts. Village health volunteers were trained to conduct door-to-door educational visits, supported by local health centers and guided by district health offices.

The Lawa Model program has implemented a multifaceted strategy encompassing chemotherapy with a single dose of praziquantel (40 mg/kg body weight), innovative and locally co-designed health education in Lawa Lake communities, ecosystem modification, and monitoring with active community participation. The WHO Collaborating Centre for Research and Control of Opisthorchiasis at Khon Kaen University offered technical and educational support for public awareness campaigns. Advocacy initiatives, such as the “*Lawa Says Goodbye to Raw Fish*” campaign, employed multimedia tools—including posters, videos, brochures, radio broadcasts, and exhibitions along with participatory workshops on fish cooking with local leaders and villagers, to raise awareness about the risks of consuming raw fish and to promote safer practices [14]. These activities have the potential to drive behavioral change within the affected community.

The “Lawa Project” also targeted schoolchildren with the launch of the “Liver Fluke-Free School” program. Over 1000 students from nine schools around Lawa Lake were screened and treated. A novel curriculum on “Liver Flukes and Cholangiocarcinoma” was developed in collaboration with local authorities [14]. The curriculum was expanded to 30 schools in Khon Kaen province and is now being rolled out nationwide as part of the national agenda to combat liver fluke infections and cholangiocarcinoma.

For animal reservoirs, we initially treated all available domestic cats and dogs in the community, followed by periodic mass deworming, education for pet owners, and collaboration with local veterinarians under the guidance of our expert veterinarian. For non-domestic animals, community-led monitoring from time to time and studies on transmission and ecological risk factors, specifically targeting *Bithynia siamensis goniomphalos*—the primary intermediate host in this area—were conducted. These efforts were complemented by environmental modifications, such as constructing a road along the lake by the local irrigation office, effectively reducing the *Bithynia* snail population by altering its natural habitat. These measures, coupled with human anthelmintic treatment, help reduce *O. viverrini* infections in the snails, leading to a decrease in fish infections.

## 5 Project outcomes

**Fig. 3:**
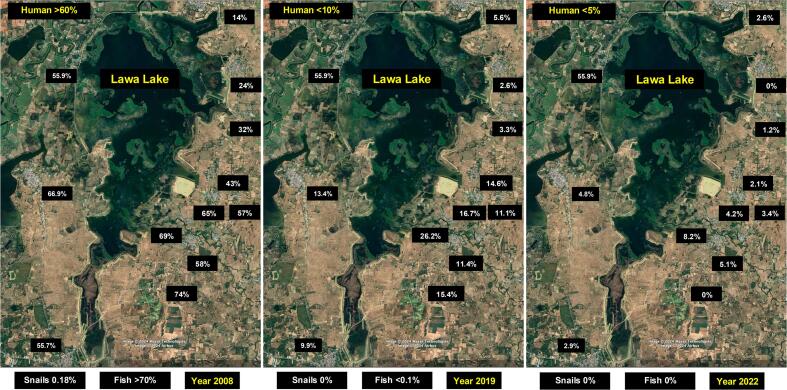
Prevalence of *Opisthorchis viverrini* infection in various villages surrounding Lawa Lake from 2008 to 2022.

The initial phase of the “Lawa Project” in the Lawa Lake community has yielded promising results. The infection rate in over 20 villages surrounding Lawa Lake has decreased significantly, from a baseline prevalence of approximately 60 % to less than 5 % in the year 2022 (Fig. 3). Remarkably, the infection rates in *Bithynia* snails and Cyprinoid fish species, the first and second intermediate hosts, are now undetectable, compared to baseline levels of up to 0.2 % and 70 %, respectively. The liver fluke infection prevalence in school children dropped from average 10 % to zero after three years of implementation. Furthermore, community knowledge about liver fluke infection and associated cancer risks has improved, leading to healthier behavioral practices. This marks an early success for the “Lawa Project” in reducing liver fluke infections in the wetland community, aligning with the Ministry of Public Health's goal to eliminate liver fluke infections and cholangiocarcinoma by 2026, with infection rates in humans, animals, and fish reduced to below 1 %.

## 6 The Lawa model achievements and beyond

The “Lawa Model” extends beyond health, driving sustainable development in the Lawa Lake community through the KKP-KKU LAWA Project, which integrates health, agriculture, alternative energy, ecology, education, and culture to holistically enhance living standards. Internationally recognized, the model was featured at the 2014 Neglected Zoonotic Diseases Conference in Geneva and adopted in the WHO's 2017 Expert Consultation on Foodborne Trematodes as a key strategy for liver fluke control. It has since been applied in liver fluke-endemic countries across the Greater Mekong Subregion. Over a decade, the Lawa Model has served as a training site for health personnel worldwide, with an online course developed during the COVID-19 pandemic, reaching scientists from over 50 countries.

## 7 Lessons learned

The Lawa Model highlights key lessons for liver fluke control, emphasizing the critical role of community engagement in fostering ownership, adherence, and sustained behavior change. Integrating multi-sectoral approaches through the EcoHealth/One Health framework effectively addresses health, environmental, and socio-economic determinants, including agricultural practices, environment management, and food safety, while involving sectors like education and governance. For low-resource settings, engagement with local authorities, specifically Subdistrict Administration Office, was crucial in securing budgetary support for annual stool examinations and intervention activities alongside with building local capacity through training ensures communities can sustain initiatives. Monitoring and evaluation enable data-driven adjustments, while culturally sensitive health education helps overcome behavioral barriers, such as raw fish consumption, promoting long-term success tailored to local contexts.

## 8 Future perspective

The future of the Lawa Model lies in scaling its application to other endemic regions in Southeast Asia and beyond through collaborations like the Mekong-Lancang Cooperation and WHO. Strengthening community engagement and integrating the model into national health strategies for neglected tropical diseases is vital [15]. Advanced technologies, such as GIS disease mapping and remote sensing, can improve surveillance, while research into treatments and health promotion remains crucial. Addressing climate change impacts and conducting long-term assessments will support adaptive strategies and highlight the value of EcoHealth/One Health approaches in global liver fluke control [16].

## 9 Conclusion

The Lawa Model represents a comprehensive, community-driven approach to controlling liver fluke infections and preventing cholangiocarcinoma. With its success in Northeast Thailand, the model serves as a prototype for other lake regions and tailored to local context, highlighting the importance of integrated EcoHealth/One Health strategies in addressing complex public health challenges. Through regional collaboration, technological innovation, and sustained government support, the Lawa Model can serve as a global One Health blueprint for the control of liver fluke infections and the prevention of associated diseases.

## Funding

This work did not receive any specific grant from funding agencies in the public, commercial, or not-for-profit sectors.

## CRediT authorship contribution statement

**Banchob Sripa:** Writing – review & editing, Writing – original draft, Conceptualization. **Sirikachorn Tangkawattana:** Writing – review & editing, Investigation. **Mingkwan Sangnikul:** Investigation.

## Declaration of competing interest

The authors declare that they have no known competing financial interests or personal relationships that could have appeared to influence the work reported in this paper.

## Data Availability

No data was used for the research described in the article.
